# Solving pickup and drop-off problem using hybrid pointer networks with deep reinforcement learning

**DOI:** 10.1371/journal.pone.0267199

**Published:** 2022-05-26

**Authors:** Majed G. Alharbi, Ahmed Stohy, Mohammed Elhenawy, Mahmoud Masoud, Hamiden Abd El-Wahed Khalifa

**Affiliations:** 1 Department of Mathematics, College of Science and Arts, Qassim University, Al Mithnab, Saudi Arabia; 2 Department of Electrical Engineering, School of Engineering and Technology, Badr University in Cairo (BUC), Cairo, Egypt; 3 Centre for Accident Research and Road Safety, Queensland University of Technology, Brisbane, Australia; 4 Department of Mathematics, College of Science and Arts, Qassim University, Al-Badaya, Saudi Arabia; 5 Department of Operations Research, Faculty of Graduate Studies for Statistical Research, Cairo University, Giza, Egypt; Hanyang University, REPUBLIC OF KOREA

## Abstract

In this study, we propose a general method for tackling the Pickup and Drop-off Problem (PDP) using Hybrid Pointer Networks (HPNs) and Deep Reinforcement Learning (DRL). Our aim is to reduce the overall tour length traveled by an agent while remaining within the truck’s capacity restrictions and adhering to the node-to-node relationship. In such instances, the agent does not allow any drop-off points to be serviced if the truck is empty; conversely, if the vehicle is full, the agent does not allow any products to be picked up from pickup points. In our approach, this challenge is solved using machine learning-based models. Using HPNs as our primary model allows us to gain insight and tackle more complicated node interactions, which simplified our objective to obtaining state-of-art outcomes. Our experimental results demonstrate the effectiveness of the proposed neural network, as we achieve the state-of-art results for this problem as compared with the existing models. We deal with two types of demand patterns in a single type commodity problem. In the first pattern, all demands are assumed to sum up to zero (i.e., we have an equal number of backup and drop-off items). In the second pattern, we have an unequal number of backup and drop-off items, which is close to practical application, such as bike sharing system rebalancing. Our data, models, and code are publicly available at Solving Pickup and Dropoff Problem Using Hybrid Pointer Networks with Deep Reinforcement Learning.

## I. Introduction

The problem of pick-up and drop-off is generally considered to be one of the most essential vehicle routing problems in our everyday lives; in fact, it is a key issue in both the Intelligent Transportation Systems and Operations Research disciplines, with widespread applications in industries such as harbor [1], airport [2], and warehousing [3]. Products must be picked up at an origin and delivered to a destination in numerous physical distribution situations. Relevant examples include transportation of disabled people, pick-up and delivery of rapid courier, delivery of various medical supplies, and so forth. Of note, however, the problem of pick-up and drop-off differs from traditional transportation challenges, as, in the former, it is obligatory to deal with issues such as priority limitations among the clients to be visited, sharing a depot with all customers; in fact, in vehicle routing problems (VRPs), a client may always have his or her own delivery site, as in intra-city express service [4] and ridesharing [5]. All these applications may readily characterize route planning as a pickup and delivery problem (PDP), which is a common kind of VRPs. Pairing and precedence connections distinguish PDPs in which a pickup point should come before the partnering delivery point. Despite extensive previous research, standard techniques, including exact and heuristic algorithms, have thus far failed to optimally solve PDPs within a short computation time because of its NP-hard nature [6].

At present, deep reinforcement learning (DRL) is being used to instantaneously master the basics in traditional heuristic approaches to solve combinatorial problems such as the Traveling Salesman Problem (TSP) and Capacitated VRP (CVRP), resulting in more alluring solutions characterized by significantly faster computing times. Inspired by previous research in these areas [7–9], in the present study, we developed a deep reinforcement learning-based model to tackle PDP. Despite some success, most DRL-based systems can manage only standard VRP with a shared delivery point. The masking method employed in previous DRL models might be intuitively extended to represent such relationships in PDP, with delivery points always masked until the pickup locations are visited. However, there remain the following two concerns that need to be considered:

1. The masking approach only affects the policy network’s output layer, and a more desired solution would enable the model to be implicitly aware of precedence relationship among nodes.
2. In contrast to a normal VRP, nodes in PDP serve many functions, such as pickup point, delivery point, and depot. Due to their intricate interplay, the decision on the next node may be more challenging.

To address these challenges, in the present study, we propose using a deep reinforcement learning-based strategy to solve PDP that integrates with Hybrid Pointer Networks [10]. The DRL’s policy network includes an encoder-decoder structure and learns to design a solution by iteratively selecting a pickup or delivery point at each time step. This approach is used in combination with a masking mechanism that adaptively filters out invalid points to guarantee feasibility and compliance with PDP constraints.

### Contributions

The contributions of the present study can be summarized as follows:

○ To address PDP, we propose a novel technique that relies on coordinate features and its constraints to enable end-to-end training for the problems with a constraint like PDP without any hand-crafted features.
○ We numerically demonstrate how such a simple approach can handle challenging problems in OP, such pickup and drop-off problems.
○ We conduct experiments on two different applications to demonstrate effectiveness of the proposed technique.
○ We conduct several ablation studies to investigate essential parameters that contribute to high performance.

The remainder of the paper is structured as follows. Section II provides a brief overview of traditional approaches and deep models for routing challenges. Section III presents an introduction to the PDP through mathematical formulation. In Section IV, we reformulate the problem in RL terms and present our DRL solution. Computational experiments and results of our analyses are presented in Section V. Finally, the results are discussed and conclusions are drawn in Section VI.

## II. Related work

**Fig 1:**
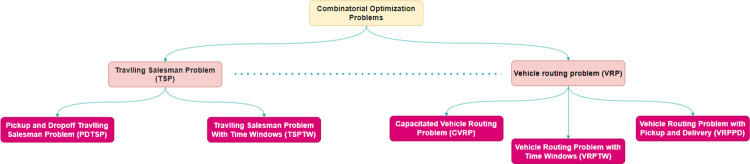
A subset of combinatorial optimization problems (COP) structure.

This section describes the tasks related to traditional PDP approaches and deep learning models of routing problems. In its simplest form, a pick-up and delivery problem (PDP) consists of a fleet of vehicles and a list of customer requests. Each request provides a starting point and a destination (i.e., a pick-up and drop-off location). Vehicles must travel in place to reach each starting point in front of the appropriate destination. This basic structure is shared by all variations of the problem. PDP is a traveling salesman problem with a restricted (multiple) solution (TSP). The PDP route is a TSP tour, and the starting point must be in front of the destination. In addition, it is necessary to maintain capacity constraints so that not to exceed the capacity of the truck. Consequently, the best TSP solution is expected to be adapted to solve the PDP. Fig 1 summarizes the COP structure. As can be seen in the figure, pickup-and-delivery traveling salesman problem (PDTSP) and traveling salesman problem with time window (TSPTW) are TSP variations with extra constraints to the traditional TSP. For instance, in TSPTW, there is another layer above the TSP formulation that sets time frames for each point, and so on. However, while there are more problems categorized under COP, for consistency considerations, we focus in the present study only on the COP that are related to our problem.

The most effective accurate TSP algorithms reported to date make extensive use of comprehensive understanding of the TSP polytope’s structure. For instance, in a previous study, a 2,392 city issue was effectively optimized using the branch-and-cut approach [11]. Furthermore, dynamic programming [12, 13], nonlinear integer programming [14, 15], and column generation [16] were used to solve variants of the fundamental problem. In addition, several heuristics were also investigated [17–19]. Many additional techniques were reviewed in Savelsbergh and Sol’s published survey [20]. Several precise techniques, such as branch- and-bound with additive bounding procedure and column generation system, were employed to handle these variations. According to some conclusions Dynamic programming was reported to work well in small-scale versions of the single-vehicle pickup and delivery problem [21]. Based on three constraints—namely, subtour elimination and precedence constraints, extended order constraints, and order matching constraints, Ruland and el. [22] proposed a branch-and-cut approach for solving PDP. Furthermore, Padberg and Rinaldi [23] devised an innovative branch-and-cut-and-price strategy to handle PDP by taking into consideration two pricing sub-problems in column formation. Despite delivering optimum answers, due to exponential complexity, appropriate procedures require a disproportionately long computation for large-scale problems. On the other hand, heuristic techniques for PDP and VRP can handle large-scale cases with a relatively good processing efficiency [24–28]. To cope with large-scale PDP, Li and Lim [29] proposed a tabu-embedded simulated annealing technique based on the shift, exchange and devised rearrange operators. Furthermore, Ropke and Pisinger [30] proposed an adaptable large neighborhood search heuristic methodology for dealing with PDP; this methodology featured a regret insertion method and six removal techniques, such as Shaw removal and cluster removal. In another relevant study, Ghilas et al. [31] proposed another adaptive big neighborhood search strategy based on break and rebuild algorithms. Starting with a greedy insertion heuristic’s initial solution, multiple efficient removal operators and insert principles were used to progressively improve the results. In addition, a hybrid three-stage heuristic approach to solve (PDTSP) was described by Hernández-Pérez, Rodríguez-Martín, and Salazar-González [32] who, after constructing early solutions with a variety of local search operators, improved the solution by combining multi-start and variable neighborhood decent heuristic techniques, as well as three shaking procedures to disturb solutions from the local minimum. However, in traditional heuristic techniques, the optimum performance, or hand-engineered rules, heavily relied on human talent and experience, which left much room for future development in solution quality. To date, deep reinforcement learning (DRL) is being increasingly used to solve several combinatorial optimization problems, such as TSP, TWTSP, and VRPs [33]. Most models based on deep reinforcement learning are defined by the policy network of the encoder and the decoder structures. Overall, an encoder projects a client (or node) to a feature embedding to extract meaningful information from the data, while a decoder solves the problem in the following two ways: either by building or improving increment.

○ In the former case, the decoder starts with an empty sequence and repeatedly selects nodes at each step in order to build the complete solution.
○ For the latter, the decoder starts with a complete initial solution, continuously selects either a specific operation candidate node or heuristic operator at each step, and maintains the current solution better than the previous solution until the termination criteria are met.

Masking strategies have always been used to obfuscate visited or invalid nodes so that each customer is visited only once. Combined with attention mechanisms and graph neural networks, DRL models can provide a higher quality solution. Another alternative that has had tremendous impact on solving COPs is Pointer Network, which solves TSP in a supervised style [34] and then is extended to reinforcement learning framework [7]. The first deep underlying architecture was developed to solve routing constraints. In addition, Pointer Network was used to solve CVRP based on reinforcement learning in the conditions where customer information is dynamic and route length is unpredictable [35]. In order to speed up the training process, the recurrent neural network (RNN) structure of the encoder was removed, as the sequence and location information is not useful for CVRP. Several previous studies (e.g., [8, 36]) presented a transformer-based architecture to provide higher quality solutions using self-attention instead of the Seq2Seq structure in both the encoder and decoder (see also [37]). A combination of local search and deep reinforcement learning were also proposed to solve both VRP and VRPTW [38]. For instance, Khalil et al. [39] used a (DQN) Deep Q-Network to train a vertices selection model that operated with a greedy search for solving TSP, where the states were represented using a Graph Neural Network, in contrast to the Seq2Seq model. GNN was also used to train supervised normalized embeddings employed to rebuild neighboring matrix of TSP graph [40]. Furthermore, reinforcement learning was used to solve the problem of electric vehicle maneuvering, and a new architecture based on decentralized learning and centralized decision making was presented [41]. To build these circuits, upon transforming the online routing problem into the in-vehicle circuit generation problem, James, Yu, and Gu [42] presented a deep reinforcement learning based solution based on the network of pointers embedded in the structure diagram. Finally, Instead of mere learning the constructive heuristic as in earlier approaches, Chen and Tian [43] and Wu et al. [44] were presented NeuRewriter and the enhanced heuristic, respectively, to learn iteratively improving an initial yet full solution with local search.

## III. Preliminary

A single vehicle and a set N of clients are used to represent the pickup and drop off problem investigated in this study. The vehicle has a starting point. Each customer *i* has a pickup and a drop off locations. The objective is to minimize the total distance traveled by the vehicle in servicing all customers.

PDP is defined through an undirected graph *G* = (*V*, *E*) where *V* is a set of nodes *i* = {0, 1,…,*m*} and *E* is a set of edges/links. *V*_0_ represents the depot node, and *V*_*i*≠0_ represents the *i*-th customer node. The vehicle has capacity *C*>0 and each customer node *V*_*i*_ has a demand *D*_*i*_. With negative demand representing pick up and positive demand representing drop off It is assumed that the depot demand *D*_0_ = 0.

Each node *V*_*i*_∈*R*^2^ is defined as 2-dimensional coordinates (*x*_*i*_, *y*_*i*_). PDP specifies a strategy in which a vehicle, originating at the depot, visits each pickup and delivery node precisely once to complete the service before returning to the depot at the end of the tour, with the goal of reducing overall travel distance. It’s worth noting that, in this example, PDP allows for consecutive pickups or deliveries, or a combination of the two, as long as the precedence constraint is fulfilled.

## IV. Methodology

In this section, we reformulate the PDP as a reinforcement learning (RL) problem, which is followed by the development of a model based on the encoder and decoder structure to learn node selection process for solution construction empowered by Hybrid pointer networks (HPN) [10].

### A. Formalization of PDP as a reinforcement learning problem

The aim of reinforcement learning is to select the best-known action for each given state, which means that the actions should be ranked and assigned corresponding values. Given that such acts are state-dependent, in essence, we should assess the value of state-action pairs. Then, in our task, a certain objective function should have been minimized. This objective function was the total distance traveled by the agent without a violation of any specified constraint.

The route-building process may be thought of as a set of decisions that can be naturally expressed in the form of RL and solved. The route-building procedure was previously described as a Markov Decision Process [45]. The state space, action space, transition rule, and reward function in the MDP were formulated as follows:

**State:** State *s*_*t*_ = *M*_*t*_ reflected the partial solution built at time step *t*, where *M*_*t*_ included all visited nodes up to step *t*, and *M*_0_ refers to the depot.

**Action:** Section a_t_ was represented as *v*_*j*_, i.e., picking node *v*_*j*_ at step *t* from the subset of the nodes allowed to visit (but not yet served).

**Transition:** The next state *s*_*t*+1_ = *M*_*t*+1_ = (*M*_*t*_;) was derived from *s*_*t*_ by picking a node at step *t*, where “;” denoted concatenation of the partial solution from the previous step with a newly picked node.

**Reward:** The reward was defined as the negative of the entire tour length computed by adding negative values of all step travel distances. As a result, the reward was denoted as shown in Eq (1).

$$R=\sum_{t=0}^{T}d_{t}$$ (1)

where *d*_*t*_ is the negative value of the incremental travel distance between *v*_*i*_ and *v*_*j*_ selected at step *t* and *t*+1, respectively. Of note, in DRL, we trained the network to learn the policy that maximized the expected sum of rewards. Therefore, in this problem context, we trained the network to learn the policy that minimized the total travel distance.

**Policy:** The stochastic policy *p*_θ_ chose a node at each time step based on the precedence criterion. This procedure was followed until all pickup-and-delivery services were completed. The ultimate result of enacting the policy was a permutation of all nodes, which specified the order of each node for the vehicle to visit, i.e., $V(\pi )=\{v_{\pi_{1}},\ldots ,v_{\pi_{T}}\}$. The probability of an output solution was factorized using the chain rule as shown in Eq (2).

$$P(V(\pi )|V)=\prod_{t=1}^{T}p_{\theta }(v_{\pi_{t}}|V,v_{\pi_{1}},\ldots ,v_{\pi_{t-1}}),$$ (2)

where *V* is the set of nodes of the instance. Decision making about the node selection based on the learnt policy *p*_θ_ was then performed.

### B. Policy Network based on Hybrid Pointer Networks (HPN)

○ **Policy’s encoder**.

A routing problem’s action space is discrete and increases exponentially with an increase of the problem size. The policy-based reinforcement technique, which consists of an actor network and a critic network [46], is frequently used to address such problems. At each time step, the actor network constructs a probability vector across all actions based on the current state and then samples an action from the allowed action set; this is recursively repeated until the termination condition is reached. The reward of the actor network is computed by accumulating the discounted reward at each step. To reduce variance, the critic network is used, as it criticizes the actions made by the actor; said differently, the critic network acts as a judger of the actor’s action, thus showing how effective (good or bad) its action was as a baseline of the actor network and calculating the baseline reward. After obtaining the actor network’s reward and the critic network’s baseline reward, the policy gradient approach [47] is used to update the parameters of the two networks, with the actor network being taught to produce higher-quality solutions.

In order to learn policy *π*, following several previous studies [10] where the HPN model was built upon the pointer networks, we built a policy network with an encoder-decoder structure. Given the features of PDP, the HPN was expected to learn the link between the nodes of various roles, allowing the precedence constraint to be captured intrinsically. As a result, the parameters in this deep architecture also corresponded to those in *π*. We then rebuilt the architecture to make it more suitable for PDP.

Technically speaking, HPN works by passing the context through two different encoders before combining the two contexts using the summing function. For instance, Stohy et al. used a GNN for context encoding in the HPN model, as the graph can extract complex relationships from a graph, and the other encoder is the transformer’s encoder [48] made up of several self-attention layers. The LSTM [49] was used in the point encoder, which encodes the currently selected point concatenated with the truck’s remaining capacity.

**Fig 2:**
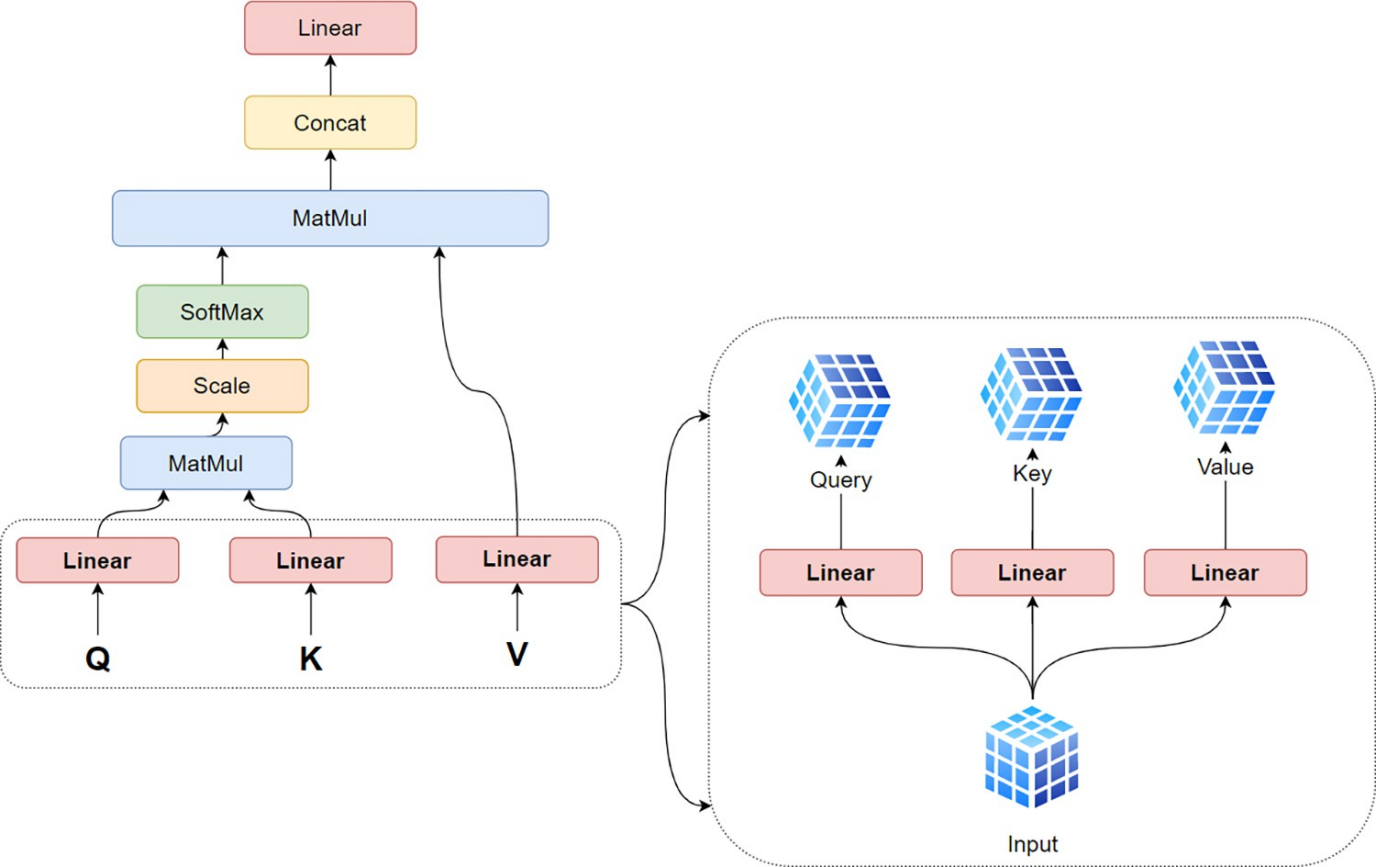
Multi-headed attention.

As can be seen in Fig 2, for the transformer’s encoder, there was the multi-head attention sublayer where we left out the depot. For simplification, $h_{i}^{l-1},i\in V$ was taken to denote the node embedding of attention layer *L*−1 (*L* ∈ {1,…,*N*}), where *i* was the node index. We then assumed *d*_*k*_, *d*_*v*_ to be the query/key dimension and *d*_*v*_ to be the value dimension, where $d_{k}=d_{v}=\frac{d_{h}}{M}$and *M* = 8 was the number of heads. For the number of heads, we followed the common and parameters used in the literature.

Given input sequence *V*, in our case, *V* was a sequence of the cities’ coordinates concatenated with the cities’ demands. In order to improve feature extraction, the self-attention mechanism learns the relationships between sequence elements to construct a representation of the sequence. Based on input *V*, three vectors—namely, query, key, and value—were constructed to capture the relationships (see Eq (3)–(7)).

$$H^{l}=softmax\left(\frac{Q^{l}K^{l^{T}}}{\sqrt{d}}\right){Val}^{l}$$ (3)

$$H^{enc}=H^{l=L^{enc}}\in R^{n\times d}$$ (4)

$$Q^{l}=H^{l}\;W_{Q}^{L}$$ (5)

$$K^{l}=H^{l}\;W_{K}^{L}$$ (6)

$${Val}^{l}=H^{l}\;W_{V}^{L}$$ (7)

where $W_{Q}^{L},W_{K}^{L}\in R^{d_{h}\;X\;d_{k}}$ and $W_{V}^{L}\in R^{d_{h}\;X\;d_{v}}$ are trainable parameter matrices, *H*^*enc*^ is a matrix containing the encoded nodes, *Q*^*l*^, *K*^*l*^ and *Val*^*l*^ are a query, key, and value of the self-attention, respectively, and *l* denotes the number of self-attention layers.

The graph encoder is the second. The context of the graph embedding layer was obtained by directly encoding the context vector or feature vector. We then simplified the equation and assumed that the graph was complete. As a result, the graph embedding layer could be written as shown in Eq (8) [50]:

$$V^{l}=\gamma \;V^{l-1}\;W_{g}+(1-\gamma )\;\varphi_{\theta }\;\left(\frac{X^{l-1}}{\left|N(i)\right|}\right)$$ (8)

where $V^{l}\in R^{N\times d_{l}},\;and\;\varphi_{\theta }:R^{N\times d_{l-1}}\to R^{N\times d_{l}}$ is the aggregation function, *γ* is a trainable parameter, $W_{g}\in R^{d_{l-1}\times d_{l}}$ is trainable weight matrix, and *N*(*i*) the adjacency set of node *i*. Next, *l* denotes the number of layers used for the graph embedding.

For the point encoder encoding the currently selected city concatenated with the truck’s remaining capacity, each city coordinates *v*_*i*_ (*i*.*e*. (*v*_*i*,1_, *v*_*i*,2_)) concatenated with *c*_*t*_ where *c*_*t*_ were taken to denote the truck’s remaining capacity at time stamp *t* the concatenated vector $\hat{v}$ is embedded into a higher dimensional vector $\hat{v}\in R^{d}$, where *d* is the hidden dimension. An LSTM then encoded the vector $\hat{v}$ for the current city *v*_*i*_. The hidden variable ${\hat{v}}_{i}^{h}$ of the LSTM was passed to both the decoder of the current stamp and the encoder of the next time stamp.

○ **Policy’s decoder**.

The decoder was built on a pointer network’s attention mechanism and outputs the pointer vector *u*_*i*_, which was then sent through a Softmax layer to generate a distribution over the following candidate points. The formulation of the attention mechanism and the pointer vector is shown in Eq (9).

$$u_{i}^{\left(j\right)}=\left\{ \begin{array}{c} A^{T}.\tanh\left(W_{r}r_{j}+W_{q}q\right)\;if\;j\neq \sigma (k),\;\forall k<j,\\ -\infty \;otherwise, \end{array}\right.$$ (9)

where Softmax normalizes the vector *u*_*i*_ to be the “attention” mask over the inputs, *u*_*i*_^(*j*)^ considered as the j-th element of the vector *u*_*i*_. *A*, *W*_*r*_ *and W*_*q*_ are trainable parameters, *q* is the query vector from the hidden state of the LSTM, r_i_ is a reference vector containing the contextual information from all cities.

The distribution policy over all candidate cities is provided in Eq (10).


$$\pi_{\theta }(v_{i}|s_{i})=p_{i}=softmax(u_{i})$$ (10)

**Fig 3:**
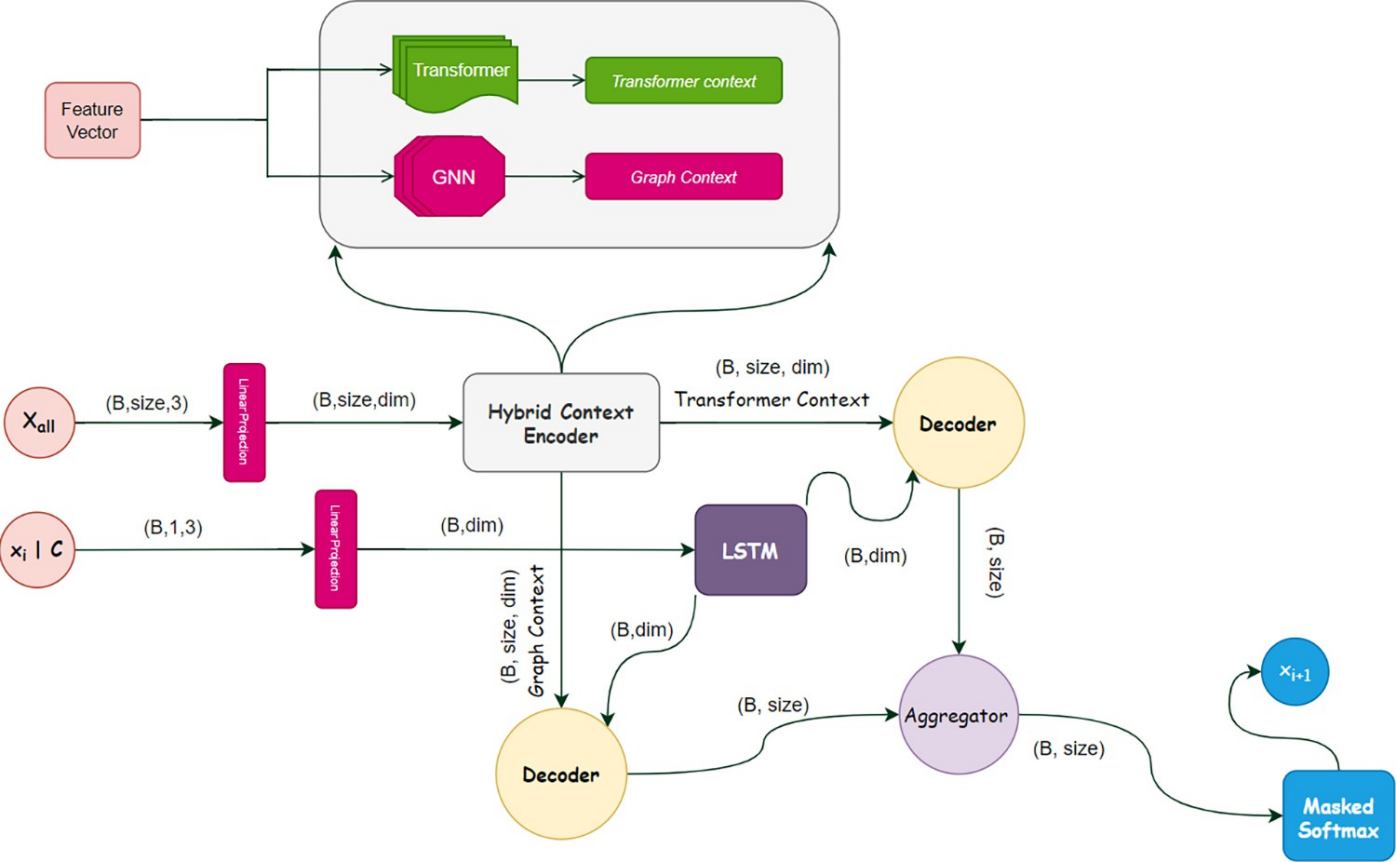
Illustration for how features flow inside HPN. Feature vector consists of thee vectors: two city’s coordinates and the cities’ demand. | means concatenate both currently selected points with the truck’s remaining capacity.

We then predicted the next visited city by sampling or choosing greedily from policy *π*_*θ*_(*v*_*i*_|*s*_*i*_).To this end, we just reconstructed that architecture to meet the requirements of the problem (see Fig 3). In our problem, we had four features *x*, *y*, *d*, and *c*, *x* and *y* were the 2-dimonational coordinates for the points, *d* represented the points’ demand and finally *c* represented the truck’s remaining capacity. As our context included *x*, *y*, and *d*, we passed these three features through the hybrid encoder and obtained two contexts: one from the GNN and the other from the transformer’s encoder. We then concatenated the truck’s remaining capacity with the currently selected point, taking the mean of the GNN context, the transformer context and projecting them into a high dimensional space. We then added both tensors together with the concatenated one and passed them through the LSTM. The architecture of our model to tackle PDP is shown in Fig 3.

### C. Training algorithm

Algorithm 1 summarizes the training of the suggested policy for solving PDP where we used the reinforcement learning approach with a roll-out baseline [8]. The policy gradient method was characterized by the following two networks:

1. The actor network drove node determination actions by making a vector of probabilities over those actions and inspected them as indicated by those probabilities to investigate the action space.
2. In the self-critic network, which is a roll-out baseline with a topology similar to that of the actor network, the reward was determined given the beginning state by selecting the node with the best likelihood to eliminate variance. After obtaining the reward of actor network *R* and the baseline reward of the critic network, the reinforcement learning technique used the policy gradient approach to adjust parameters of two networks.

Furthermore, when the performance of the latter was considerably better according to the results of a paired t-test on a specified number of batches, the parameters of the critic network were substituted with those of the actor network. Policy P was trained to identify higher-quality solutions by upgrading the two networks.

**Algorithm 1. REINFORCE with Rollout Baseline [8]**

1: Input: number of epochs E, steps per epoch T, batch size B, significance α

2: Init *θ*, *θ*^*BL*^ ← *θ*

3: For *epoch* = 1,…,*E* do

4: For *step* = 1,…,*T* do

5: *s*_*i*_ ← RandomInstance () ∀*i* ∈ {1,…,*B*}

6: *π*_*i*_← SampleRollout (*s*_*i*_, *p*_*θ*_) ∀*i* ∈ {1,…,*B*}

7: $\pi {}_{i}^{BL}$ ← GreedyRollout $\left(s_{i},p{}_{\theta }^{BL})\;\forall i\in \{1,\ldots ,B\right\}$

8: $\nabla L\leftarrow \sum_{i=1}^{B}L(\pi_{i})-L(\pi {}_{i}^{BL})\;\nabla_{\theta }\;log\;p_{\theta }(\pi_{i})$

9: θ ← Adam (θ, *∇*ℒ)

10: End for

11: If OneSidedPairedTTest (*p*_*θ*_, $p{}_{\theta }^{BL}$) **<** α then

12: *θ*^*BL*^ ← *θ*

13: End if

14: End for

## V. Experimentation and computation analysis

In this section, we report the results of simulations performed to validate the effectiveness of the proposed model to address PDP. A truck left the warehouse and made only one stop for each customer, i.e., the pickup location had to be visited before the delivery one. The goal was to minimize the overall trip. Overall, PDP is an NP-hard problem characterized by growing computation complexity with an increase of the problem size.

### Experimental settings

As mentioned above, PDP was defined through an undirected graph *G* = (*V*, *E*) where node *i* = {0, 1,…,*m*} was re-presented by features *n*_*i*_. The index *i* = 0 represented the depot node, and *i* > 0 represented the *i-th* customer node. The vehicle had capacity *D* > 0 and each customer node *i* = {1,…,*m*} had a demand δ_I_, 0 < *δ*_*i*_ < *D*. We assumed that the depot demand δ_0_ = 0. Both depot and customer nodes were randomly generated inside the unit square [0, 1] × [0, 1]. We generated 21 and 51 (the first node was the depot) for instances with a size of m = 20 and 50; due to memory constrain, we did not go through size 100. We used Kaggle’s Tesla P100 with 16 GB of memory. The corresponding vehicle capacities were 30 and 40, respectively. The customer node demands were uniformly sampled from {1,2,…,9}. We normalized the customer node demand to [0, 1] by the truck’s max capacity through $\delta_{i}^{norm}=\frac{\delta_{i}}{truck\;capcity}$ The input, masks, and decoder context vectors for PDP were adjusted as follows:

1. **Input**: Handling PDP needed only to expand the node feature *n*_*i*_ to a four-dimensional input $n_{i}^{\prime }$ that included the normalized demand $\delta_{i}^{norm}$, node feature *n*_*i*_ (nodes’ coordinates), and the nodes’ Euclidian distance from depot (see Eq (11)).
$$n_{i}^{\prime }=n_{i}||\delta_{i}^{norm}||{Euc}_{depot}$$ (11)
  where || represents concatenation.
2. **Vehicle remaining capacity update**: We masked out the customer nodes that were served, so there was no need to update the needs of served customer nodes. The decoder selected the customer node *π*_*t*_ at timestep t, and the remaining capacity of the vehicle was represented by $D_{t}^{\prime }$. We assumed that the vehicle started at the depot when the decoding timestep *t* = 1 and the vehicle was zero loaded (with the remaining vehicle capacity $D_{1}^{\prime }=0$). The remaining vehicle capacity was updated using Eq (12).
$$D_{1}^{\prime }=\left\{ \begin{array}{l} D_{t-1}^{\prime }-\delta_{\pi_{t}}^{norm}\;\pi_{t}=i,i\in \{1,\ldots ,m\}\\ zero\;\pi_{t}=0 \end{array}\right.$$ (12)
3. **Decoder context vector**: The context vector *c*_*t*_ of the decoder at timestep *t* ∈ {1,⋯,*m*}, where m was the number of nodes without depot, of three components: hybrid embedding $\bar{x}$, embedding of node *π*_*t*_ and vehicle remaining capacity $D_{t-1}^{\prime }$ (see Eq (13)):
$$c_{t}=\left\{ \begin{array}{l} \bar{x}+W_{x}(x_{\pi_{t-1}}||D_{t-1}^{\prime })\;t>1\\ \bar{x}+W_{x}(x_{0}||D_{t-1}^{\prime })\;t=1 \end{array}\right.$$ (13)
  where *W*_*x*_ is a trainable parameter.
4. **Mask update mechanism**: For PDP, our mask consisted of the following two parts: (1) the customer node mask and (2) the depot node mask. For the customer node mask, we masked out:
  ○ Customer node that was served;
  ○ Any pickup city if its demand plus the remaining capacity exceeded the truck’s limit (1);
  ○ When the demand of a customer node exceeded the remaining capacity of the vehicle. That is, customer node *i*’s attention coefficient *u*_*i*,*t*_ = −∞ when $\delta_{i}^{norm}>D_{t-1}^{\prime }$ or $i\neq \pi_{t},\forall t^{\prime }<t,i\in \{1,\ldots ,m\}$.
  ○ For the depot mask, as the depot would not be the next chosen node when the vehicle left the depot, the depot node 0’s attention coefficients *u*_*i*,*t*_ = −∞ for *t* = 1 or *π*_*t*−1_ = 0

Next, in order to test our model capability and to generalize through different settings, we then did the following two separate applications:

1. In the first application, considered for bike sharing system BBS, we assumed that all demands summed to zero, and our goal was to satisfy the demands as much as we could, and the truck would leave the depot with a zero capacity.
2. In the second application, we assumed more randomness in demands and randomized the demands while balancing the number of the pickup points with the drop-off one.

The points of the stations and clients (pickup and drop-off) nodes were then arbitrarily and freely created involving a 2-layered uniform circulation in the scope of 0 and 1, where the distance between two hubs was determined in view of Euclidean space. Hyperparameters used during training are summarized in Table 1.

**Table 1:** Hyperparameters used for training both experiments.

Parameter	Value	Parameter	Value
Graph embedding layer	3	Learning rate	1 x 10^−4^
Transformer encoder layer	6	Batch size	512
Feed-forward dim	512	Training steps	2500
Optimizer	Adam	Tanh clipping	10
Epochs	100	Number of hidden unites for embedding	128

**i. Bike Sharing Systems (BBS)**.

In order to ensure customer satisfaction and system effectiveness, it is essential to rebalance BSS bike distribution [51]. In previous extensive research on redistribution, numerous efficient methods that maintain an equilibrium of bikes at each station have been proposed.

The three forms of BSS rebalancing include static, dynamic, and incentivized rebalancing. A fleet of trucks is frequently used to redistribute bikes in static and dynamic rebalancing models. First, static rebalancing, alternatively referred to as SBRP, is usually performed at night or during periods of low demand. This is so because SBRP assumes that the number of bikes needed at each station remains constant or shows only light fluctuations. Second, the rebalancing outcome in the Dynamic Bicycle Repositioning Problem (DBRP) is influenced by bike movement, which has a substantial impact on bike demand at each station. Consequently, solving DBRP requires accurate demand forecasting. Third, incentivized rebalancing promotes users to participate in system rebalancing by advising modest changes to their intended itinerary via control signals, which provides alternative routes that promote rebalancing, or even paying users for returning bikes to a station. In recent years, BSSs have outgrown the need for stations/docks, with users becoming able to deposit and pick up bikes anywhere within designated municipal boundaries. This sort of bike-sharing, also known as a free-floating bike-sharing system (FFBSS), has several important advantages over traditional BSSs, such as cheaper capital costs and lower risk of bike theft. The system’s efficient rebalancing is critical to its success [52].

The results on BBS21 and BBS51 where the truck’s capacity will be 30 and 40, respectively, are summarized in Table 2. As can be seen in the table, our model achieved a low tour length as compared with the other two models with a large margin. For the sack of clarity and to demonstrate effectiveness of our approach, we used previously proposed models [34, 50] that handled the traveling salesman problem (TSP) with our settings.

**Table 2:** Our result on BBS21 and BBS51, obj denotes for the total tour length and the Time denotes for the time for evaluation the result averaged over 10k instances.

	BBS21 Max Vehicle’s Capacity = 30 Initial load = zero		BBS51 Max Vehicle’s Capacity = 40 Initial load = zero	
Method	Obj	Time	Obj	Time
Pointer Networks Greedy	4.642	1s	7.01	2s
GPN Greedy	4.625	1s	7.00	3s
HPN Greedy	**4.22**	1s	**6.34**	4s

**Fig 4:**
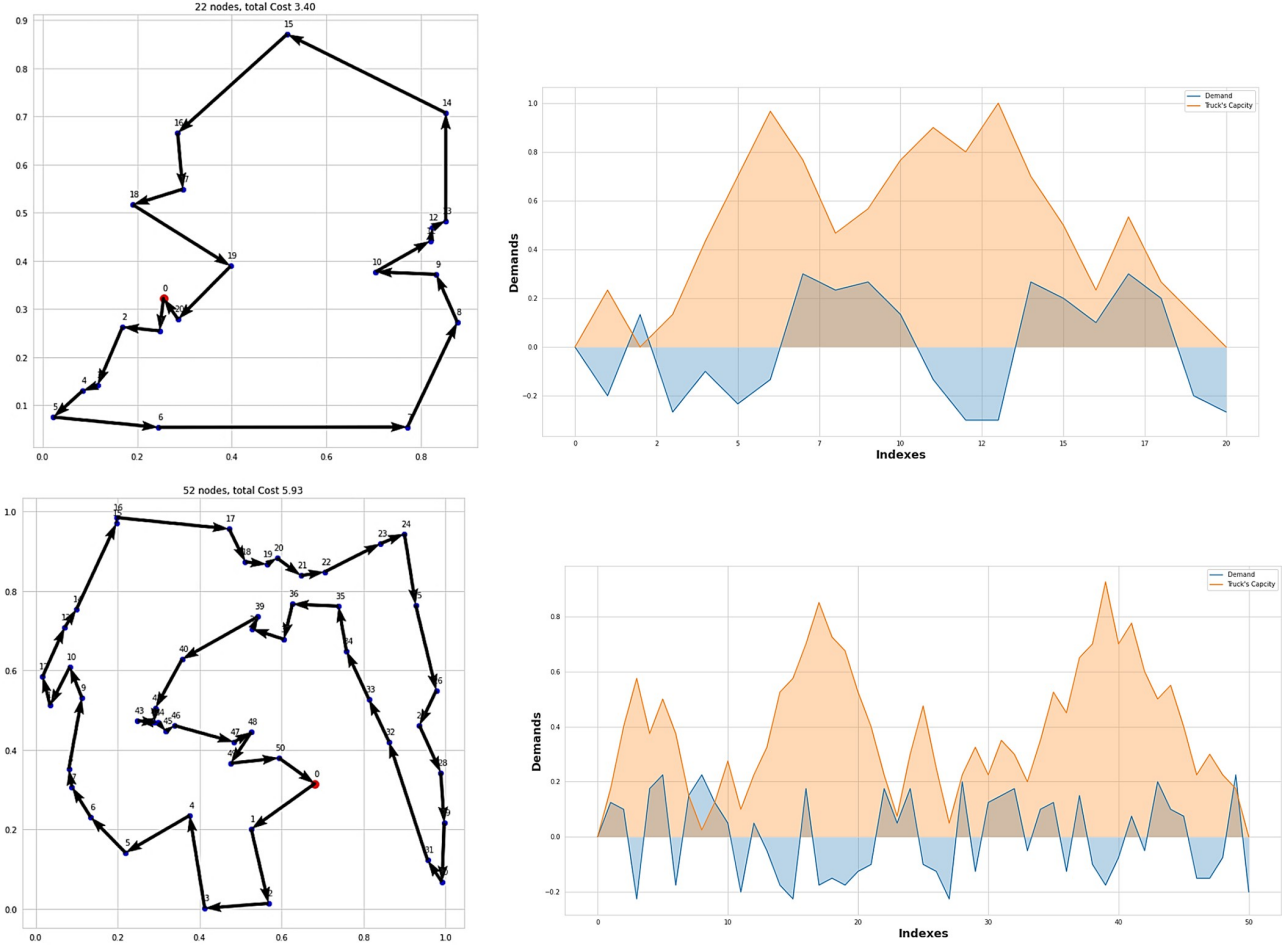
Sample instances for BBS21 and BBS51, with each point labeled with its demand and the vehicle remining capacity once the vehicle served this point.

Several representative instances for BBS21 and BBS51 are shown in Fig 4. As can be see from the the line plot in Fig 4, at the end of the tour, the truck’s capacity returned to zero, and the overall truck’s capacity during the tour did not exceed its limit (1). This results demonstarted feasibility of the proposed solution.

ii. **PDP with random demands**.

In our second application, we tested our methedology against more randomness in generation demands. To this end, we generated equal drop-off and pickup points; yet, we did not ensure that their sum would be equal to zero. In order to esnure finding a feasible soluation for each batch, we also assumed that the agent was allowed to visit the depot and refill half of the truck during to gurantee. Our results on PDP21 and PDP 51 where the truck’s capacity was 30 and 40, respectively, are summarized in Table 3.

**Table 3:** Results on PDP21 and PDP51.

	PDP21 Max Vehicle’s Capacity = 30 Initial load = zero		PDP51 Max Vehicle’s Capacity = 40 Initial load = zero	
Method	Obj	Time	Obj	Time
Pointer Networks Greedy	4.863	1s	7.403	2s
GPN Greedy	4.856	1s	7.436	3s
HPN Greedy	**4.30**	1s	**6.48**	4s

**Fig 5:**
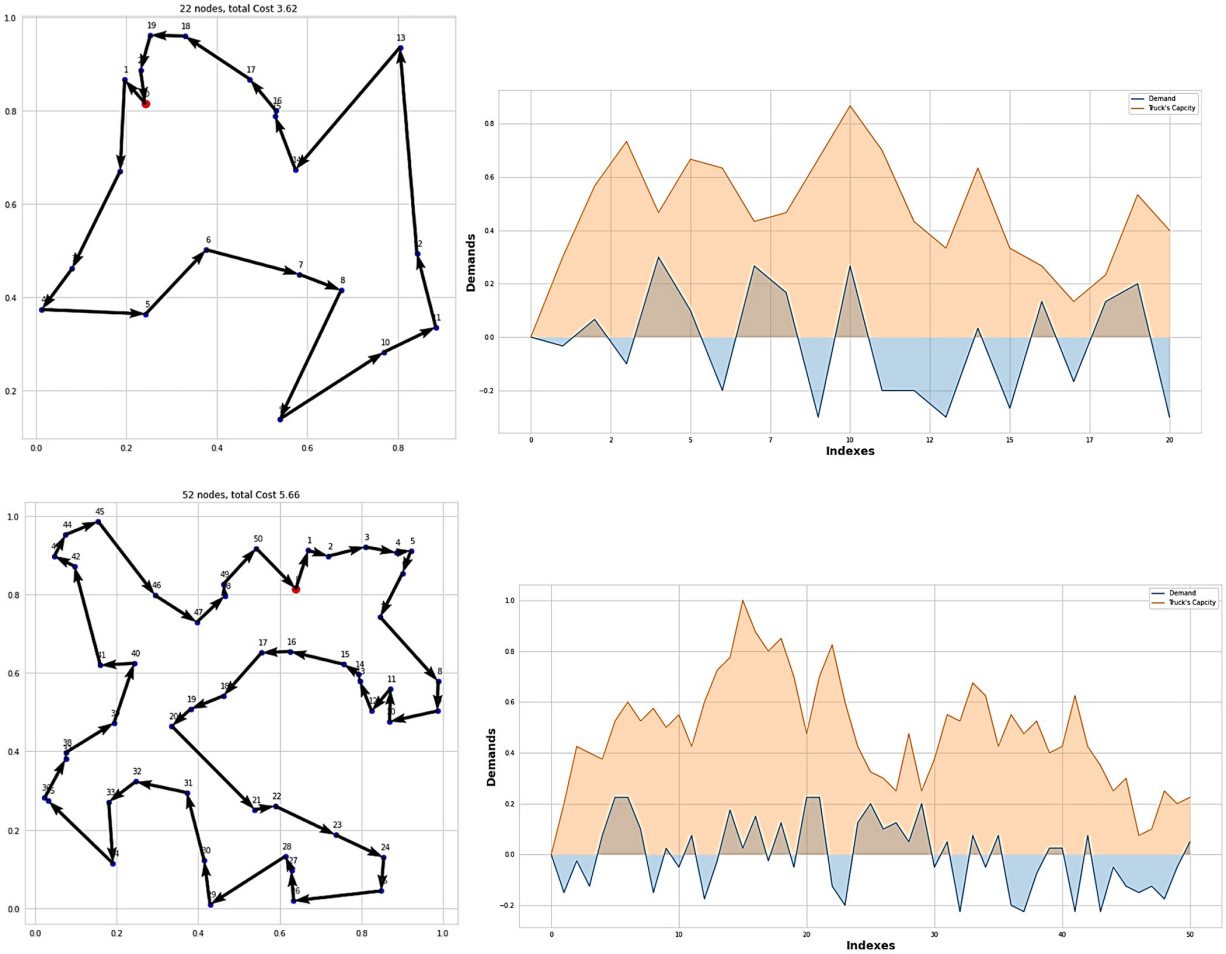
Sample instances for PDP21 and PDP 51, with each point labeled with its demand and the vehicle remining capacity once the vehicle served this point.

As can be seen in Table 3, our model had the lowest tour length with a large margin for both sizes as compared to GPN and Pointer Network. Fig 5 shows some representative instances for PDP21 and PDP51. As can be seen from the line spot in Fig 5, the truck’s capacity did not execeed its limit, so the solution can be evaluated as feasible.

**iii. Large instances**.

Given that handling huge instances is a significant challenge, when dealing with real-world applications, a high number of points is to be expected, whi is the criterion that makes one model better than others. In our experiments, we sought to generalize our approach for large instances. In order to achieve the best potential generalization from our model, we used a simple approach of diffusing the generalization capability throughout our model’s parameters. We then trained our model on instances of size 50 and validated our training on instances of size 500 to fine-tune the model’s parameters for bigger instances; our assumption was that the sum of the demands would tend to be equal to zero. Upon setting the batch size of the trainig data to 128 and the validation data to 64, we trained our model only for 4 epochs to avoid overfitting. Finally, in order to avoid memroy limitation, we decreased the number of hidden unites to 64. A summary of our training hyperparameters during training of large instances is provided in Table 4.

**Table 4:** Hyperparameters used for training of large instances.

Parameter	Value	Parameter	Value
Graph embedding layer	3	Learning rate	1 x 10^−4^
Transformer encoder layer	6	Batch size	512
Feed-forward dim	512	Training steps	2500
Optimizer	Adam	Tanh clipping	10
Epochs	4	Validation size	500
Number of hidden unites for embeddings	64	Validation Batch size	32

**Fig 6:**
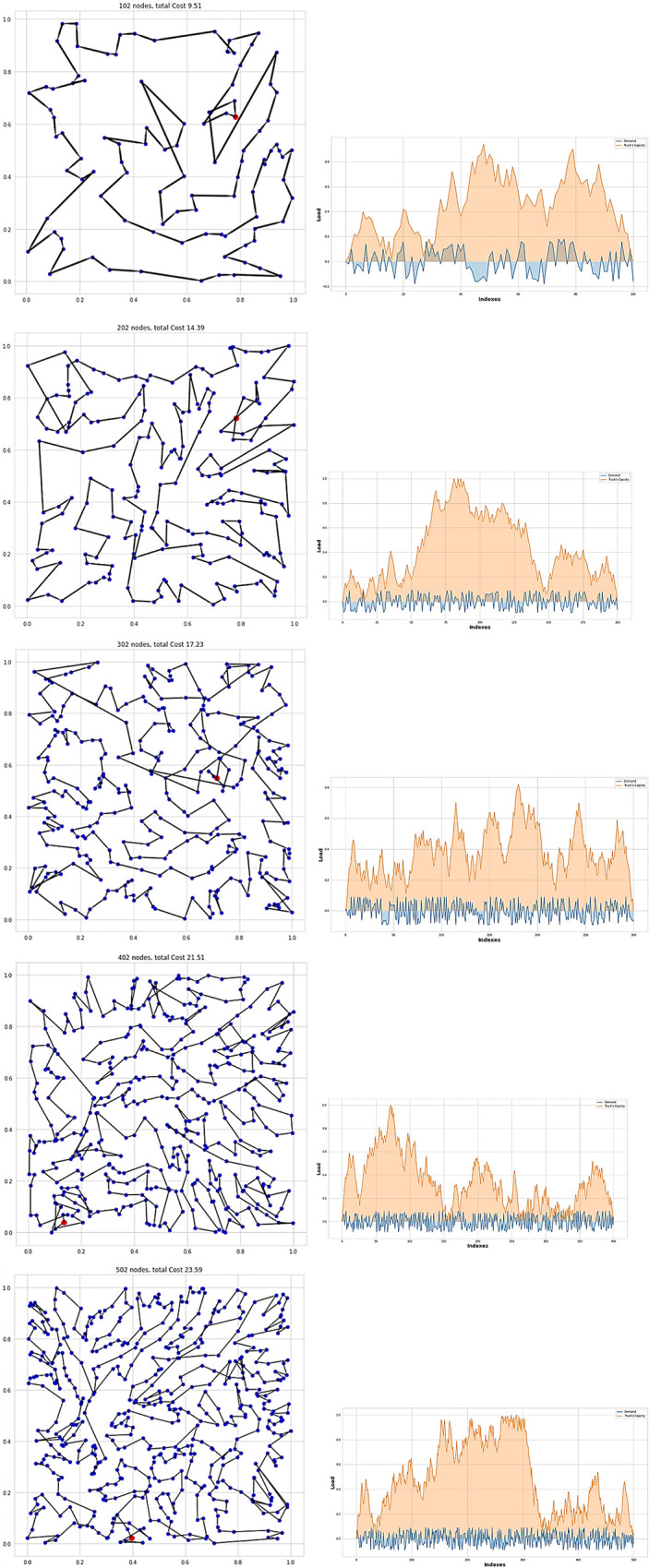
Sample tour for BBS101, BBS201, BBS301, BBS401 and BBS501.

Table 5 shows the result of BBS101,BBS201,BBS301,BBS401,and BBS501. Our assumption was that the truck’s max capacity for all of these instances was 40. Due to the memory constraint, we averaged only over 10k intances. Fig 6 illustrates several simple tours for BBS101,BBS201,BBS301,BBS401, and BBS501.

**Table 5:** Results of BBS101, BBS201, BBS301, BBS401, and BBS50.

	BBS101 Max Vehicle’s Capacity = 40 Initial load = zero		BBS201 Max Vehicle’s Capacity = 40 Initial load = zero		BBS301 Max Vehicle’s Capacity = 40 Initial load = zero		BBS401 Max Vehicle’s Capacity = 40 Initial load = zero		BBS501 Max Vehicle’s Capacity = 40 Initial load = zero	
Method	Obj	Time	Obj	Time	Obj	Time	Obj	Time	Obj	Time
GPN Greedy	11.02	24s	18.144	30s	25.058	40s	31.788	48s	38.35	55s
HPN Greedy	**10.88**	28s	**17.12**	38s	**23.07**	45s	**29.29**	50s	**34.74**	58s

Note. The results averaged over 10K instances.

## VI. Ablation studies

In this section, we present the results of our ablation studies and explain our design decisions in the light of recent literature.

### a. Model choice

While a trade-off between speed and accuracy may be achieved by adjusting the model selection, we discovered that the HPN has a superior Encoder-Decoder combination to deal with complicated input relations. Therefore we opted to tweak this model to deal with PDP.

### b. Result analysis

The total demand for a standard bike-sharing system (BBS) tended to be zero. We drew both the truckload and the point’s requests during the journey. Following intuition, for our process to be correct, the truckload should have stayed inside its. Our findings revealed that exceeding the truck limit was impossible, and the model attempted to balance loading during the route, which resulted in a loading plot with a line that rarely exceeded 80% of the truck capacity. Then, in order to see evaluate training stability, we increased the model’s burden and randomly generated demands for both the pickup and drop-off points; however, to ensure visibility of the solution, we added a more relaxing option for the agent and let the agent return to the depot and refill half of the truck capacity. The results showed that all model’s solutions were feasible, including the condition with the capacity margin. Although we experienced difficulties when applying this approach to large-scale instances due to memory constraints, using the validation trick during model training yielded good results with only 16G of RAM. It can be speculated that increasing batch size during training large scale of instance would provide more stable training process and better results than those we obtained.

## VII. Conclusion

In this study, we proposed a new approach to handle pickup and drop-off on a small and large scale that combines a hybrid pointer network (HPN) with deep reinforcement learning. Applying this technique to two separate types of PDP, we found that our technique can successfully resolve these sorts of difficulties and reach state-of-the-art outcomes. While, for large instances, our model was still struggling for an optimal solution, it demonstrated a better generalization capability as compared with GPN and pointer networks. Accordingly, considering the challenge of determining the optimal solution for large points should be a primary emphasis in further research. In our future work, we will seek to establish a rigorous approach to enhance the quality and speed of large-scale issue solutions, which would then lead to higher model generalization.
